# Continuous Noninvasive Remote Automated Blood Pressure Monitoring With Novel Wearable Technology: A Preliminary Validation Study

**DOI:** 10.2196/24916

**Published:** 2022-02-28

**Authors:** Michael H McGillion, Nazari Dvirnik, Stephen Yang, Emilie Belley-Côté, Andre Lamy, Richard Whitlock, Maura Marcucci, Flavia K Borges, Emmanuelle Duceppe, Carley Ouellette, Marissa Bird, Sandra L Carroll, David Conen, Jean-Eric Tarride, Prathiba Harsha, Ted Scott, Amber Good, Krysten Gregus, Karla Sanchez, Pamela Benoit, Julian Owen, Valerie Harvey, Elizabeth Peter, Jeremy Petch, Jessica Vincent, Michelle Graham, P J Devereaux

**Affiliations:** 1 School of Nursing McMaster University Hamilton, ON Canada; 2 Population Health Research Institute Hamilton, ON Canada; 3 McGill University Montreal, QC Canada; 4 Faculty of Health Sciences McMaster University Hamilton, ON Canada; 5 Department of Medicine Faculty of Nedicine Université de Montréal Montréal, QC Canada; 6 Research and Innovation Hamilton Health Sciences Hamilton, ON Canada; 7 Cardiac and Vascular Intensive Care Unit Hamilton Health Sciences Hamilton, ON Canada; 8 University of Toronto Toronto, ON Canada; 9 Centre for Data Science and Digital Health Hamilton Health Sciences Hamilton, ON Canada; 10 Institute for Health Policy, Management, and Evaluation University of Toronto Toronto, ON Canada; 11 University of Alberta Edmonton, AB Canada

**Keywords:** validation study, continuous vital signs monitor, continuous non-invasive blood pressure monitoring, wearable, blood pressure, monitoring, validation, mHealth, vital sign, biosensor, accuracy, usability

## Abstract

**Background:**

Wearable continuous monitoring biosensor technologies have the potential to transform postoperative care with early detection of impending clinical deterioration.

**Objective:**

Our aim was to validate the accuracy of Cloud DX Vitaliti continuous vital signs monitor (CVSM) continuous noninvasive blood pressure (cNIBP) measurements in postsurgical patients. A secondary aim was to examine user acceptance of the Vitaliti CVSM with respect to comfort, ease of application, sustainability of positioning, and aesthetics.

**Methods:**

Included participants were ≥18 years old and recovering from surgery in a cardiac intensive care unit (ICU). We targeted a maximum recruitment of 80 participants for verification and acceptance testing. We also oversampled to minimize the effect of unforeseen interruptions and other challenges to the study. Validation procedures were according to the International Standards Organization (ISO) 81060-2:2018 standards for wearable, cuffless blood pressure (BP) measuring devices. Baseline BP was determined from the gold-standard ICU arterial catheter. The Vitaliti CVSM was calibrated against the reference arterial catheter. In static (seated in bed) and supine positions, 3 cNIBP measurements, each 30 seconds, were taken for each patient with the Vitaliti CVSM and an invasive arterial catheter. At the conclusion of each test session, captured cNIBP measurements were extracted using MediCollector BEDSIDE data extraction software, and Vitaliti CVSM measurements were extracted to a secure laptop through a cable connection. The errors of these determinations were calculated. Participants were interviewed about device acceptability.

**Results:**

The validation analysis included data for 20 patients. The average times from calibration to first measurement in the static position and to first measurement in the supine position were 133.85 seconds (2 minutes 14 seconds) and 535.15 seconds (8 minutes 55 seconds), respectively. The overall mean errors of determination for the static position were –0.621 (SD 4.640) mm Hg for systolic blood pressure (SBP) and 0.457 (SD 1.675) mm Hg for diastolic blood pressure (DBP). Errors of determination were slightly higher for the supine position, at 2.722 (SD 5.207) mm Hg for SBP and 2.650 (SD 3.221) mm Hg for DBP. The majority rated the Vitaliti CVSM as comfortable. This study was limited to evaluation of the device during a very short validation period after calibration (ie, that commenced within 2 minutes after calibration and lasted for a short duration of time).

**Conclusions:**

We found that the Cloud DX’s Vitaliti CVSM demonstrated cNIBP measurement in compliance with ISO 81060-2:2018 standards in the context of evaluation that commenced within 2 minutes of device calibration; this device was also well-received by patients in a postsurgical ICU setting. Future studies will examine the accuracy of the Vitaliti CVSM in ambulatory contexts, with attention to assessment over a longer duration and the impact of excessive patient motion on data artifacts and signal quality.

**Trial Registration:**

ClinicalTrials.gov NCT03493867; https://clinicaltrials.gov/ct2/show/NCT03493867

## Introduction

### Background

Intraoperatively, continuous hemodynamic monitoring is the standard of care for patients undergoing surgery [1]. Continuous monitoring of patients’ vital signs in the operating room (ie, blood pressure [BP], heart rate, respiratory rate, blood oxygen saturation [SpO_2_], core body temperature, and electrocardiogram [ECG]) facilitates immediate recognition of hemodynamic instability and patient deterioration [1]. In contrast, once patients are transferred to surgical wards, their vital signs are assessed only periodically [2]. Hospital policies typically dictate that nursing staff assess patients’ vital signs every 4 hours to 12 hours on surgical wards [2-6]. Patients are then discharged home routinely without surveillance [2]. Such infrequent in-hospital monitoring, followed by no monitoring at home, presents a danger to surgical patients. Cumulative published data support that deteriorations in patients’ physiologic status in hospital, for example, often go undetected [7,8], conferring risk for hemodynamic compromise and serious postoperative adverse events (eg, hypotension leading to myocardial ischemia, stroke, and death).

New remote automated monitoring (RAM) technologies that enable continuous acquisition of physiologic data from biosensors; transmission, integration, and syntheses of multiple data sources to indicate patient status; as well as real-time alerts to clinicians have the potential to revolutionize the science of RAM [2]. Major developments in the field over the last decade include (1) the evolution of RAM systems capacity for semi-automatic (ie, clinician-promoted) discrete measurement of vital signs to fully automatic continuous measurement of vital signs; (2) the development of ultra-lightweight, unobtrusive sensors that facilitate unencumbered patient ambulation; and (3) the incorporation of more powerful microprocessors that enable higher sampling frequencies and, ultimately, higher fidelity signal inputs for increased precision of early adverse event detection [2,9,10]. These advancements are now seeing the commercial availability of a few noninvasive systems [11,12] that are capable of incorporating combinations of a number of vital signs parameters and related metrics, including heart rate, respiratory rate, skin temperature, SpO_2_, BP, and movement.

Although significant progress has been made, continuous RAM systems are not yet in routine use in clinical care. A number of tactical and feasibility-related barriers remain, related to signal transmission, range, and speed; duration of power supply; as well as cybersecurity concerns [2,10]. At the clinical care level, a key barrier to advancing RAM has been the need to rely on systems that employ traditional methods for measuring BP noninvasively [2]. Such methods include the use of a sphygmomanometer with manual measurements by auscultation of Korotkoff sounds [13] or palpatory methods [14] and the derivation of automatic measurements through oscillometry [13]. These methods provide discrete or interval-based measurements with a pneumatic cuff typically situated on the brachial or radial arteries.

A challenge with systems that feature intermittent, pneumatic cuffs for the measurement of noninvasive BP is that they can be uncomfortable for patients and infeasible for longer-term patient monitoring [2]. Moreover, reliance on pneumatic cuffs does not help to overcome the problem of episodic vital sign measurement on surgical wards [2]. It is crucial that reliable, continuous, noninvasive blood pressure (cNIBP) measurement be achieved—while clinically important hypotension has been shown to have significant population-attributable risk for postoperative death and stroke, prolonged episodes of hypotension (and hypertension) are often missed in the context of intermittent BP monitoring [6-8].

Recent technologies for cNIBP measurement have emerged that utilize volume-clamp and arterial applanation tonometry methods [15]. Although these cNIBP methods have resulted in clinically accurate medical devices, they are limited in terms of portability and ambulatory use, shorter durations of application due to patient discomfort, and high cost [15]. The pursuit of cNIBP methods that provide seamless integration into a patient’s daily activities and that offer a low-cost alternative while delivering clinical-grade BP metrics is a current focus for the biomedical engineering and RAM communities [10]. Cloud DX has developed one such device called the Vitaliti continuous vital signs monitor (CVSM), which supports the derivation of cNIBP through fundamental principles of biomechanics and pulse wave velocity [2].

### Objectives

In accordance with standards set forth by the International Organization for Standardization (ISO 81060-2:2018) [16], we sought to establish the accuracy of Vitaliti CVSM cNIBP measurements versus gold standard invasive continuous arterial BP measurements in postsurgical patients. A secondary objective was to examine the usability of the Vitaliti CVSM with respect to perceived patient acceptance.

### Vitaliti Continuous Vital Signs Monitor

**Figure 1:**
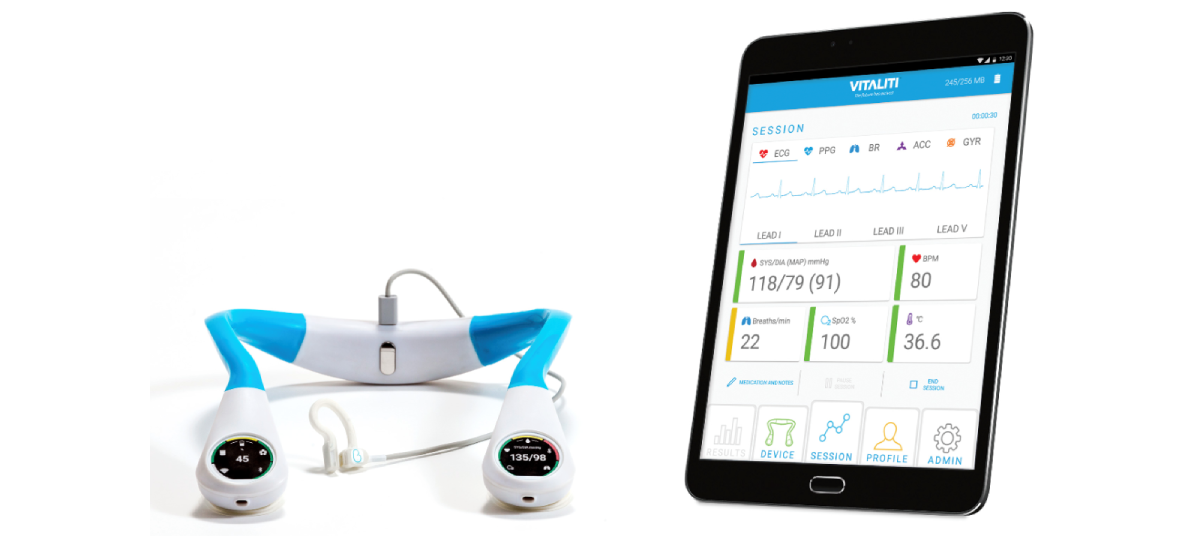
The Vitaliti continuous vital signs monitor and user interface.

The Vitaliti CVSM [2,17] (Figure 1) is a wearable CVSM that can continuously and noninvasively measure 5-lead ECG, heart rate and heart rate variability, respiration rate, temperature (infrared sensor applied to the ear), SpO_2_, and cNIBP [2]. See Multimedia Appendix 1 for details on Vitaliti CVSM donning, device configuration and features, and clinical workflow including calibration procedure.

## Methods

### Testing Authorization and Measurement Standards Requirements

The verification testing portion of this study received an investigational testing authorization (STP-VIT-002) for Class II medical devices from Health Canada. Study setting, inclusion criteria, and methods were in compliance with ISO 81060-2:2018 requirements [16], as described in the following sections.

#### ISO 81060-2-2018 Requirements

For the demographic requirements, ISO 81060-2:2018 [16] stipulates that cNIBP testing must include a minimum of 15 patients and that 30% of the sample are male and 30% are female. In addition, those included for verification testing were to meet the following required proportions for baseline BP ranges [16]:

- At least 10% shall have a reference systolic blood pressure (SBP) ≤100 mm Hg (13.33 kPa).
- At least 10% shall have a reference SBP ≥160 mm Hg (21.33 kPa).
- At least 10% shall have a reference diastolic blood pressure (DBP) ≤70 mm Hg (9.33 kPa).
- At least 10% shall have a reference DBP ≥85 mm Hg (11.33 kPa).

In keeping with ISO restrictions for special populations [16], patients who were pregnant or experiencing cardiac arrhythmias were to be excluded.

For the accuracy requirements, according to the ISO standard [16], one determination of cNIBP measurement represents the average of one 30-second interval for a given patient position. To ensure equal weighting of BP measurements across participants, the ISO standard also requires that *no more than 10* BP measurements be included per patient. Thus, for each test session, 3 separate 30-second determinations were calculated per patient for each position for both the arterial catheter reference and the Vitaliti CVSM. Errors of each measurement determination were calculated. If the determination of the Vitaliti CVSM was within 1 (±) SD of the determination of the arterial catheter, the error of that determination equaled 0. If any SBP or DBP determination from the Vitaliti CVSM was outside of 1 (±) SD of the corresponding arterial catheter determination, then the error for that determination equaled the upper or lower limit of the arterial catheter reference measurement minus the Vitaliti CVSM determination [16].

All errors of valid, paired BP determinations (included participants only) were then used to calculate the experimental mean and SD of errors for SBP and DBP. If the mean of the errors of determination was not greater than 5 mmHg and the SD of the error was not greater than 8 mmHg, then the Vitaliti CVSM device was determined to be compliant with ISO guidelines [16].

Bland-Altman plots [18] were generated to visualize agreement between arterial catheter and Vitaliti CVSM mean BP measurements and inspect the bias (ie, mean error) and distribution of errors of determination within 95% limits of agreement (ie, ±1.96 SD).

### Setting and Participants

This study required comparison of the Vitaliti CVSM to a gold-standard comparator for continuous BP measurement. We therefore required access to patients with an invasive arterial catheter for hemodynamic monitoring. Recruited participants provided written, informed consent and included patients 18 years of age or older who underwent cardiac surgery and were admitted for immediate postoperative recovery in the Hamilton Health Sciences (Hamilton General Hospital site) Cardiac Surgical Intensive Care Unit (ICU) with an arterial line in situ. The ICU setting was chosen given that arterial lines for the continuous measurement of SBP, DBP, and mean arterial pressure are the standard of care in this setting. Moreover, based on operating room schedules, the cardiac surgical unit had predictable patient flows, allowing for planning and efficient execution of study procedures. The study coordinating center was the Population Health Research Institute (PHRI) in Hamilton, Ontario.

Given minimal ISO requirements [16] for participant numbers and prespecified baseline BP ranges, we targeted a maximum recruitment of 80 participants for verification and acceptance testing. We intentionally oversampled given the high likelihood of labile hemodynamic status in postoperative cardiac surgery patients. Based on clinical experience, we anticipated that abrupt changes in baseline BP, or other aspects of physiologic status, would preclude moving forward with testing procedures for some participants. Given the complexity of the clinical setting, we also oversampled in anticipation of interruptions to study procedures (eg, immediate patient care needs, emergencies) and technical challenges with respect to data downloads and intersystem comparisons in the context of real-time cNIBP monitoring.

### Procedures and Data Collection

**Figure 2:**
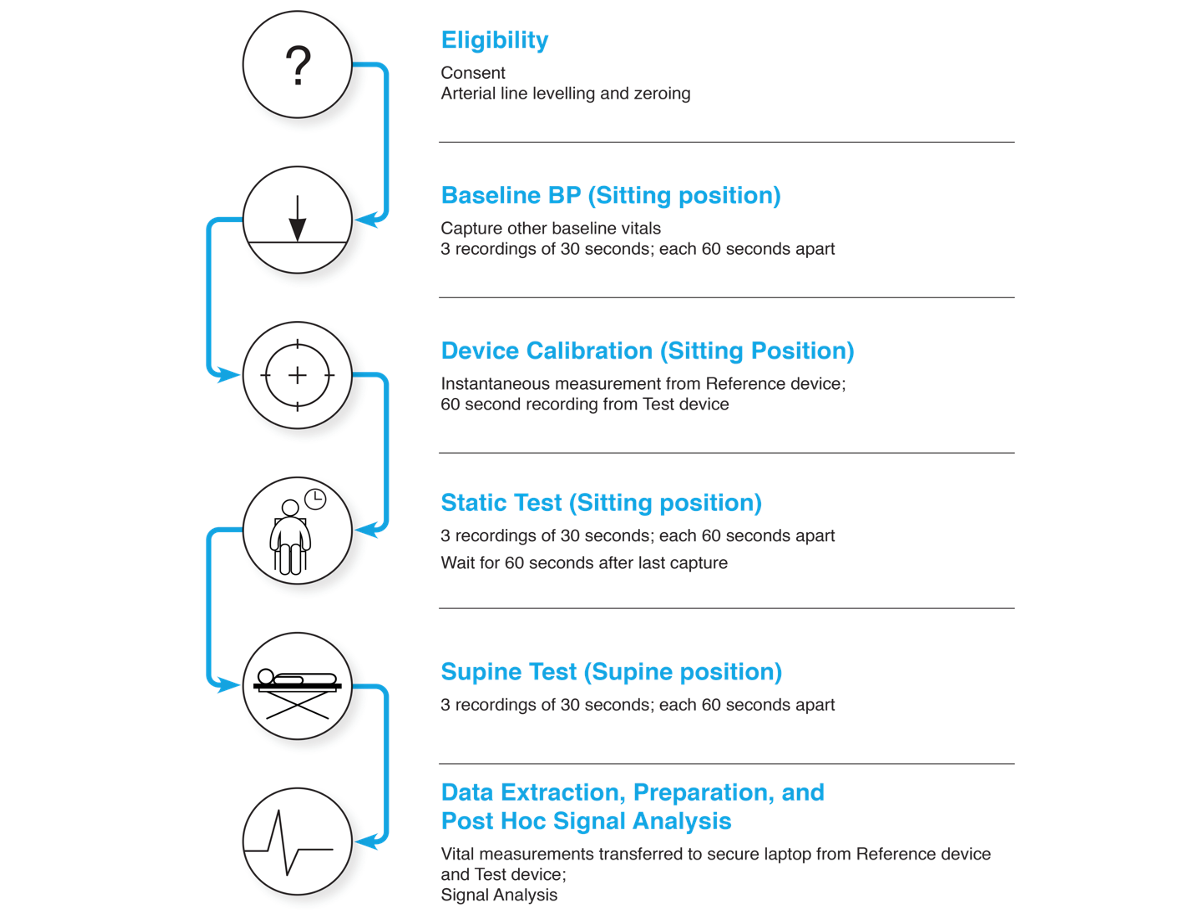
Study flow diagram.

Study flow is depicted in Figure 2. Patients expected to fulfill eligibility criteria were first approached and invited to participate by the ICU nurse educator. Those interested in hearing more were then approached by study personnel to obtain written, informed consent and collect baseline demographic information.

### Continuous Blood Pressure Measurement Requirements and Data Collection

#### ISO Guidance

ISO [16] stipulates that, in the context of cNIBP testing where gold-standard comparator devices and test devices are cuffless, cNIBP determinations measured during 30-second intervals are considered equivalent to a single determination with a traditional cuff-based sphygmomanometer. This guidance was used to capture baseline BP recordings as well as all ISO [16] test recordings, as described in the following sections.

#### Baseline Blood Pressure Recording

Baseline BP was determined from the gold-standard ICU arterial catheter. The patients’ ICU nurse first levelled and zeroed the ICU arterial catheter transducer (TruWave disposable pressure transducer; Edwards Lifesciences, Irvine, CA) to achieve consistent reference measurements and to negate the influence of external atmospheric pressure on BP recordings. Per ISO requirements [16], 3 BP recordings were then taken to establish each patient’s baseline BP category. Patients were assisted by the ICU nurse into a seated position in bed and asked to sit quietly. Three BP recordings, lasting 30 seconds each, were taken by the research assistant; each of these readings was taken 60 seconds apart. The mean value of these readings was defined as the patient’s baseline BP, and this value was logged according to the appropriate ISO category [16], as applicable. Those patients who did not meet one of the prespecified baseline BP category requirements were immediately excluded, and their participation was discontinued.

#### Vitaliti CVSM Donning Process and Setup

Per manufacturer instructions, the research assistant placed the Vitaliti CVSM around the patient’s neck and positioned the collar to be flush with the neck and shoulders. The flexibility of the device allowed for positioning of the collar and contact electrodes on the chest, such that surgical site dressings or ICU equipment and tubing were unencumbered. A disposable sheath (for infection control purposes) was placed on the tip of the earpiece, which was then positioned in the patient’s ear. The research assistant then used a tablet to access Vitaliti companion software, in order to conduct a systems check. This check included ensuring proper positioning and contact of all sensors on the patient, as well as real-time visualization of the capture of all biometrics and physiologic wave forms.

#### Vitaliti CVSM Calibration

Following baseline BP assessment and equipment setup, the Vitaliti CVSM was calibrated against the reference arterial catheter. Patients were again asked to sit still and refrain from movement or talking during this step. The research assistant first recorded an instantaneous reference BP reading from the arterial catheter and registered this value into the Vitaliti application on the tablet. Next, the Vitaliti system captured and analyzed the patients’ vital metrics and physiological signals for a period of 60 seconds, in order to calibrate against the reference measurement. At the conclusion of this calibration step, the Vitaliti system analyzed the recorded data to ensure consistent signal quality and that there were limited movement artifacts. If the Vitaliti software indicated to the research assistant that the calibration was unsuccessful, the procedure was repeated.

#### Test Blood Pressure Recordings: Static and Supine Positions

Simultaneous cNIBP readings from the arterial catheter and the Vitaliti CVSM were first captured with the patient in the seated, static position (in bed). These simultaneous measurements were captured for a period of 10 minutes, without interruption. Following this procedure, the patient was assisted by the ICU nurse into the supine position, in order to achieve a change in posture for continued measurement. Another 10 minutes of simultaneous cNIBP recordings were then captured.

### Data Extraction and Preparation for Analysis

At the conclusion of each test session, captured cNIBP measurements were extracted from the arterial catheter ICU monitor device using MediCollector BEDSIDE [19] data extraction software. Vitaliti CVSM measurements were extracted by connecting the device USB Type C port to a secure study laptop with a cable. Custom Python scripts were provided by Cloud DX to extract measurements from the Vitaliti CVSM at 1-second time-stamped intervals, for comparison against the arterial catheter data. Both devices were synchronized to ensure time alignment in post-signal processing.

**Figure 3:**
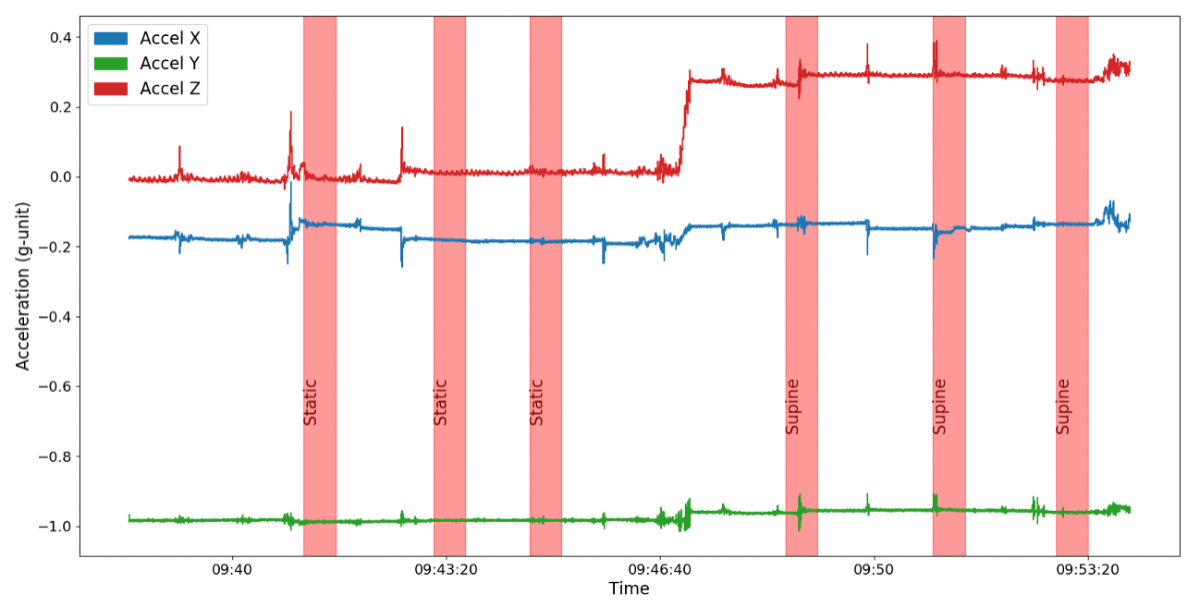
Tri-axial accelerometer data showing static and then supine patient positions with overlaid 30-second measurement intervals.

The 10-minute intervals of cNIBP recordings with patients in seated (static) and supine position alignments were verified with Vitaliti CVSM accelerometer and gyroscope data collected during the test period (Figure 3). Per ISO requirements [16], for each patient, we isolated 3 separate 30-second intervals of cNIBP measurements for seated and supine positions; each of these determinations was at least 1 minute apart. Each 30-second interval also had to feature uninterrupted cNIBP measurements without any measurement loss from either the arterial catheter or the Vitaliti CVSM. The 30-second intervals selected for analysis were extracted to allow for 2-minute transition periods between patient positions in order to ensure stable measurements.

### Vitaliti Device—Perceived User Acceptance

Human factors testing is utilized to evaluate if a medical device can support users in the intended environment for all critical tasks [20]. To provide an assessment of elements of human factors and usability of the Vitaliti CVSM from the patient perspective, an exit interview was conducted with patients by the clinical team, and an online survey was completed to capture their responses. A customized questionnaire was developed in keeping with regulatory guidance provided by the Food and Drug Administration (FDA) [20] for applying human factors and usability engineering to medical devices. This questionnaire consisted of 13 questions to establish user acceptance of the Vitaliti CVSM with respect to comfort, ease of application, sustainability of positioning, and aesthetics; possible responses to each item ranged from “strongly disagree” to “strongly agree.” See Multimedia Appendix 2 for the questionnaire.

### Data Analyses

#### Demographic Characteristics and User Acceptance Ratings

Descriptive statistics were used to summarize participants’ demographic characteristics and user acceptance ratings. The distribution of patients across baseline Association for the Advancement of Medical Instrumentation (AAMI)-ISO BP categories [16] was summarized. Analyses were performed using SAS/STAT (release 9.4) statistical analysis software. Prior to analyses, Python was used to prepare BP recordings captured by the reference arterial catheter and the Vitaliti CVSM for post hoc signal analyses.

#### Post Hoc Signal Analyses

In accordance with AAMI-ISO guidelines [16], all cNIBP measurements, in either the static or supine position and for any given participant, were excluded if (1) the invasive reference SBP had a range ≥20 mm Hg (2.67 kPa) or (2) the invasive reference DBP had a range ≥12 mm Hg (1.6 kPa). All cNIBP recordings were evaluated against these criteria. All participants with data meeting these constraints were removed from validation analyses.

Two additional assessments of signal quality were performed on the physiological data captured by the Vitaliti CVSM. First, tri-axial accelerometer and gyroscope data were reviewed to identify any test sessions with an excessive amount of movement that impacted the quality of photoplethysmography and ECG signals (metrics vital to the derivation of cNIBP). An activity index based on accelerometer data [21], defined as the time derivative of acceleration, was used to evaluate the amount of movement by each patient during test sessions. This index reflects the combined impact of the rate at which a patient’s acceleration measurements change with respect to time in 3 perpendicular planes of movement. An average value of the activity index was calculated for all activity throughout the static and supine positions. An equivalent activity index has been used for mobile application–based activity monitoring and a wellness motivation system for senior adults [21]. Vitaliti CVSM activity intensities were empirically derived during data collection; a critical threshold of 2.4 gravities per second (g/s) represented “vigorous patient activity,” equivalent to patient movement on a treadmill with a speed of 4.5 miles to 5 miles per hour [21]. Activity levels above this threshold were deemed to have negatively impacted signal quality for the purposes of our validation analysis. Data from participants with an index score ≥2.4 g/s were removed from the study [21].

Second, all ECG recordings were examined for noise, given that poor signal quality would introduce false positive R peaks in the QRS complex, which could affect the performance of the BP algorithm [22]. Cases were identified where the ECG signal quality was low, such that R peaks could not be reliably determined in the resultant signal; these cases were subsequently removed prior to validation analysis [22].

#### Validation Analyses

According to the ISO standard [16], one determination of cNIBP measurement represents the average of one 30-second interval for a given patient position. Thus, for each test session, 3 determinations were calculated for each position, for both the arterial catheter reference and the Vitaliti CVSM. Errors of each measurement determination were calculated. If the determination of the Vitaliti CVSM was within 1 (±) SD of the determination of the arterial catheter, the error of that determination equaled 0. If any SBP or DBP determination from the Vitaliti CVSM was outside of 1 (±) SD of the corresponding arterial catheter determination, then the error for that determination equaled the upper or lower limit of the arterial catheter reference measurement minus the Vitaliti CVSM determination [16].

All errors of valid, paired BP determinations (included participants only) were then used to calculate the experimental mean and SD of errors for SBP and DBP. If the mean of the errors of determination was not greater than 5 mm Hg and the SD of the error was not greater than 8 mm Hg, then the Vitaliti CVSM device was determined to be compliant with ISO guidelines [16]. Bland-Altman plots [23] were generated to visualize agreement between arterial catheter and Vitaliti CVSM mean BP measurements and inspect the bias (ie, mean error) and distribution of errors of determination within 95% limits of agreement (ie, ±1.96 SD).

## Results

### Demographics

**Figure 4:**
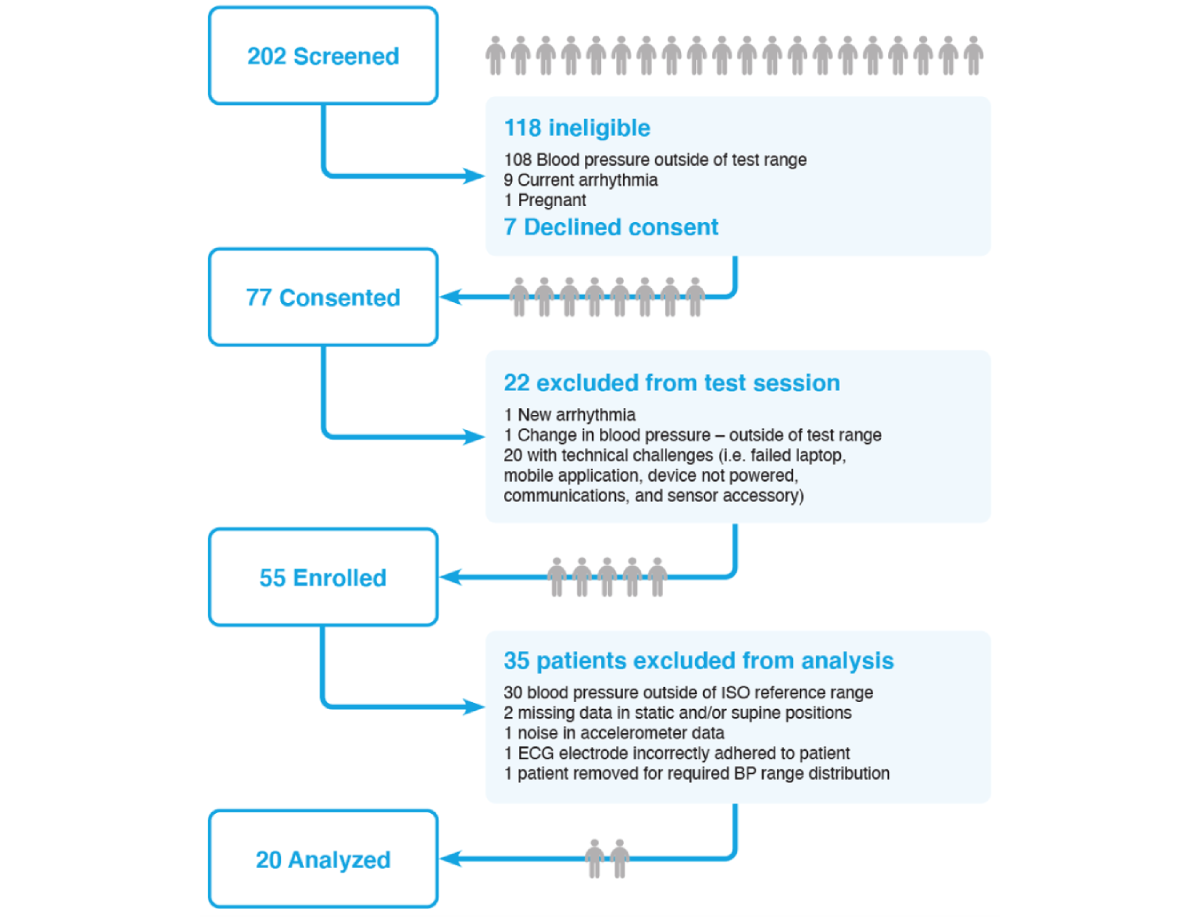
Derivation of the study sample. BP: blood pressure; ECG: electrocardiogram; ISO: International Organization for Standardization.

Derivation of the sample is presented in Figure 4. In total, 202 patients were screened for inclusion in the cardiac ICU between June 2018 and October 2019. Of these, 118 were ineligible due to baseline BPs outside of ISO requirements [16], current arrhythmia (ie, atrial fibrillation), or pregnancy; 7 patients declined participation; and 77 eligible patients consented to participate. Of the 77 eligible patients who consented to participate, an additional 22 were excluded due to technical challenges that precluded completion of the test sessions, shift in BP outside of study requirements, and development of a new arrhythmia (ie, atrial fibrillation) prior to the start of testing procedures. Technical challenges included wireless communication problems, data extraction software failures, reference device data transfer problems, and sensor disconnections. A total of 55 patients were included for validation testing procedures. Of these, 35 patients (64%) were male, and 20 patients (36%) were female; the mean age was 64 (SD 11.5) years (Table 1). Most patients had undergone cardiac surgery (33/55, 60%) including coronary artery bypass grafting or aortic valve repair.

**Table 1:** Patient characteristics.

Patient characteristics		Values for entire sample (n=55)	Values for subgroup of patients included in the validation (n=20)
Age (years), mean (SD)		64 (11.5)	64.7 (10.9)
**Sex, n (%)**			
	Male	35 (64)	11 (55)
	Female	20 (36)	9 (45)
**BMI (kg/m^2^), n (%)**			
	<18.5 (underweight)	2 (4)	0 (0)
	18.5-24.9 (healthy)	12 (22)	5 (25)
	25.0-29.9 (overweight)	18 (33)	8 (40)
	30.0-34.9 (obese I)	9 (16)	3 (15)
	35.0-39.9 (obese II)	7 (13)	1 (5)
	≥40 (obese III)	6 (11)	2 (10)
	Unavailable^a^	1 (2)	1 (5)
**Cardiac surgery, n (%)**			
	Total	33 (60)	15 (75)
	Coronary artery bypass grafting (CABG)	25 (76)^b^	13 (87)^c^
	Aortic valve repair or replacement (AVR)	6 (18)^b^	1 (7)^c^
	Other	2 (6)^b^	1 (7)^c^
**Vascular surgery, n (%)**			
	Total	11 (20)	3 (15)
	Open abdominal aortic aneurysm repair	1 (9)^d^	1 (33)^e^
	Aorto-femoral bypass	2 (18)^d^	0 (0)^e^
	Axillo-femoral bypass	1 (9)^d^	0 (0)^e^
	Femoral-femoral bypass	1 (9)^d^	0 (0)^e^
	Other	6 (55)^d^	2 (67)^e^
Other type of surgery, n (%)		11 (20)	2 (8)

^a^Patient height, weight, or BMI data unavailable from clinical record.

^b^n=33.

^c^n=15.

^d^n=11.

^e^n=3.

### Post Hoc Signal Analysis

Based on signal analysis, the data for an additional 30 patients were excluded due to arterial catheter reference SBP (≥20 mm Hg) or DBP (≥12 mm Hg) ranges falling outside of ISO requirements during each 30-second measurement interval in both static and supine testing positions. The data for 2 additional patients were excluded due to missing data segments (for either the arterial catheter reference or Vitaliti CVSM) that precluded analysis. Missing data were caused by unforeseen interruptions in data transmission related to excessive movement of the arterial catheter transducer or accidental disconnection or displacement of the Vitaliti CVSM earpiece or ECG electrodes. Finally, the data for the last patient recruited with baseline BP within the normal range were excluded, as this group would have been over-represented for the required ISO BP range distributions. Following signal analysis, the cNIBP data of 20 patients were included for validation analyses. Table 1 presents the demographic characteristics of all 55 patients enrolled, as well as the demographic characteristics of the 20-patient subgroup (of the 55 enrolled patients) who was included in validation analyses.

In accordance with the AAMI-ISO guidelines [16], of the 20 patients whose data were included for validation analyses, a minimum of 30% were male, and a minimum of 30% were female. Baseline arterial catheter cNIBP measurements also spanned high and low systolic and diastolic ranges, with at least 10% of readings falling into each AAMI-ISO prespecified category (See Table 2).

**Table 2:** Blood pressure distribution of patients included in data analysis (n=20).

Characteristic		Results, n (%)	
**Entry ISO^a^ BP^b^ range**			
	Normal: SBP^c^ >100 mm Hg and <160 mm Hg and DBP^d^ >70 mm Hg and <85 mm Hg	11 (55)	
	SBP ≤100 mm Hg	2 (10)	
	DBP ≤70 mm Hg	6 (30)	
	SBP ≥160 mm Hg	3 (15)	
	DBP ≥85 mm Hg	2 (10)	
**Sex**			
	Male	11 (55)	
	Female	9 (45)	

^a^ISO: International Organization for Standardization.

^b^BP: blood pressure.

^c^SBP: systolic blood pressure.

^d^DBP: diastolic blood pressure.

### Validation of Continuous Noninvasive Blood Pressure Measurements

For each of the 20 patients included in the final analysis, 3 determinations were calculated for both the reference arterial catheter and Vitaliti CVSM within each position (static and supine), resulting in a total of 60 average SBP and DBP measurements. The average time elapsed from calibration to first measurement in the static position was 133.85 seconds (2 minutes 14 seconds). The average time elapsed from calibration to first measurement in the supine position was 535.15 seconds (8 minutes 55 seconds). With respect to delimitation of the total validated time frame across patient positions, (1) the minimum and maximum times elapsed from calibration to first measurement in the static position were 14.0 seconds and 1392.0 seconds, respectively, and (2) the minimum and maximum times elapsed from calibration to last measurement in the supine position were 575.0 seconds and 2274.0 seconds, respectively.

**Figure 5:**
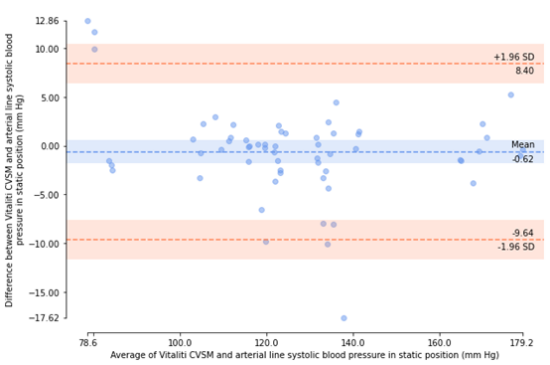
Bland-Altman plot of systolic blood pressure determinants from the Vitaliti continuous vital signs monitor (CVSM) versus the arterial line in the static position.

**Figure 6:**
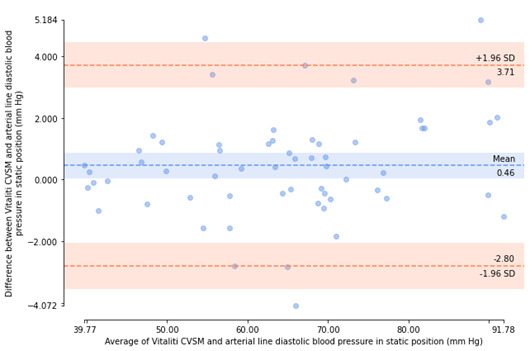
Bland-Altman plot of diastolic blood pressure determinants from the Vitaliti continuous vital signs monitor (CVSM) versus the arterial line in the static position.

**Figure 7:**
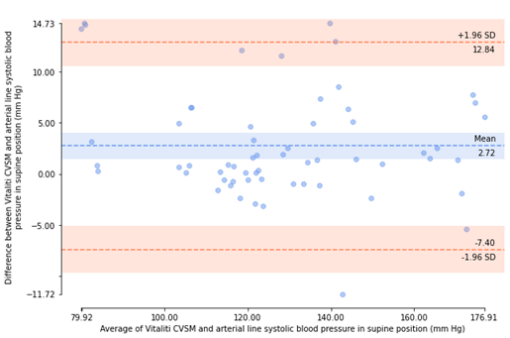
Bland-Altman plot of systolic blood pressure determinants from the Vitaliti continuous vital signs monitor (CVSM) versus the arterial line in the supine position.

**Figure 8:**
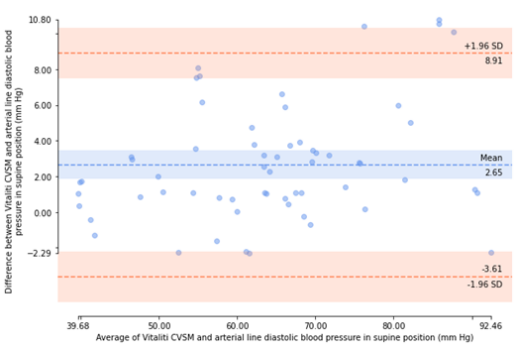
Bland-Altman plot of diastolic blood pressure determinants from the Vitaliti continuous vital signs monitor (CVSM) versus the arterial line in the supine position.

The errors of determination between the 2 devices were calculated, as described in the Methods section. Bland-Altman plots [18], illustrating agreement between the arterial catheter reference and the Vitaliti CVSM for each of these errors of determination by patient position (static and supine), are presented in Figures 5-8. The mean (horizontal axis) and errors (vertical axis) of each determination are presented, along with the mean error and limits of agreement (±1.96 SD). In the static position, Bland-Altman plots illustrated a mean error of determinations of –0.62 mm Hg and 95% limits of agreement of –9.64 mm Hg to 8.40 mm Hg for SBP measurements and a mean error of determinations of 0.46 mm Hg and 95% limits of agreement of –2.80 mm Hg to 3.71 mm Hg for DBP measurements. In the supine position, Bland-Altman plots revealed a greater mean error of determinations (2.72 mm Hg) and 95% limits of agreement of –7.40 mm Hg to 12.84 mm Hg for SBP measurements and mean error of determinations of 2.65 mm Hg and 95% limits of agreement of –3.61 mm Hg to 8.91 mm Hg for DBP measurements.

Per ISO requirements, Table 3 summarizes the errors of determination for SBP and DBP measurements in the static and supine positions. The overall means of the errors of determination for the static position were –0.621 (SD 4.640) mm Hg for SBP and 0.457 (SD 1.675) mm Hg for DBP. Errors of determination were slightly higher for the supine position, at 2.722 (SD 5.207) mm Hg for SBP and 2.650 (SD 3.221) mm Hg for DBP. These results indicate compliance with the ISO standard [16], which stipulates that errors of determination should not exceed 5 mmHg and that the SD of the error not exceed 8 mm Hg.

**Table 3:** Summary of the errors of determination for systolic blood pressure (SBP) and diastolic blood pressure (DBP) measurements in static and supine positions.

Position			SBP		DBP	
**Static position**						
	Number of observations	60		60		
	Mean of the errors (mm Hg)	–0.621		0.457		
	SD of the errors (mm Hg)	4.640		1.675		
**Supine position**						
	Number of observations	60				60
	Mean of the errors (mm Hg)	2.722				2.650
	SD of the errors (mm Hg)	5.207				3.221

### Patient Usability Feedback

**Figure 9:**
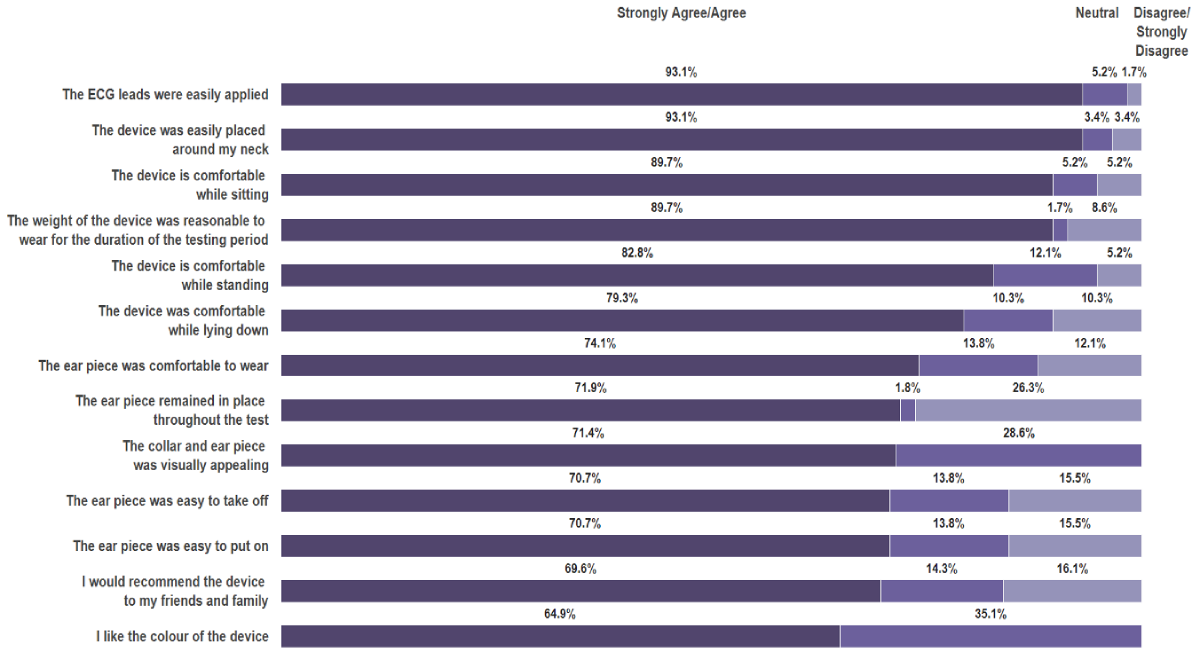
Usability feedback. ECG: electrocardiogram.

The responses from the 58 patients who responded to the human factors and usability feedback questionnaire are summarized in Figure 9. Questions related to ease of donning Vitaliti and device accessories yielded high acceptance ratings, with 54 (54/58, 93%) agreeing or strongly agreeing. Responses regarding device comfort in multiple positions were also positive, ranging from 52 (52/58, 90%) participants responding with strongly agree/agree to 40 (40/58, 75%) participants responding with strongly agree/agree. Questions regarding aesthetics of the device provided more neutral responses (range: 16/58, 28% to 20/58, 35%), indicating that these aspects were of lesser importance for most. The item regarding the sustainability of the positioning of the earpiece yielded the greatest amount of negative responses (disagree/strongly disagree: 15/58, 26%), indicating that dislodgment of this sensor during testing was an issue for just over 25% of respondents. 

## Discussion

### Principal Findings

This validation study addressed the accuracy and usability of the Vitaliti CVSM for the measurement of cNIBP in an ICU setting. Device performance was evaluated based on the ISO 81060-2:2018 standard [16] for clinical investigation with a reference invasive arterial catheter BP monitor. This standard is accepted by the FDA and Health Canada. Human factors validation testing was also performed to evaluate if the Vitaliti CVSM was acceptable to users. A voluntary exit interview was conducted to capture participants’ usability feedback. In total, 55 participants completed the testing procedures and the exit interview. Data for 20 of these participants were included for validation analyses.

When comparing the accuracy of the Vitaliti CVSM to a gold-standard reference, we found errors of determination of –0.621 (SD 4.640) mm Hg for SBP and 0.457 (SD 1.675) mm Hg for DBP in the static (seated) position. Errors of determination were slightly higher when patients were supine, at 2.722 (SD 5.207) mm Hg for SBP and 2.650 (SD 3.221) mm Hg for DBP. These results indicate compliance with the ISO standard [16], which stipulates that errors of determination should not exceed 5 mm Hg and that the SD of those errors should not exceed 8 mm Hg.

This validation study also demonstrated a high degree of usability in terms of perceived patient acceptance of the Vitaliti CVSM throughout the test procedures and positions (ie, static and supine). Displacement of the earpiece was the most negative aspect of patient experience. Earpiece displacement was caused by our use of an off-the-shelf disposable ear sheath to cover the light emitting diode (LED) sensor in order to comply with hospital infection requirements. In future work, the Vitaliti CVSM will require custom earpiece sheathing with improved fit in order to provide more secure mounting of the LED sensor and improved patient comfort.

### Comparison With Prior Work

A few comparable studies have assessed the accuracy of wearable technologies for continuous vital sign measurement in hospital. A pilot study by Weenk et al [11] (n= 20) investigated the use of 2 wearable technologies, ViSi Mobile and HealthPatch, to continuously measure vital signs (ie, heart rate, respiration rate, SpO_2_, BP) of patients admitted to an internal medicine and surgical ward; patients’ vital signs were measured continuously for 2 days to 3 days. A comparison was performed between continuous ViSi Mobile and HealthPatch measurements and nurses’ manual vital sign observations entered into an electronic medical record. Results demonstrated that, in general, nurses’ manual vital sign observations correlated well with paired instances of continuous vital sign measurements. Data artifacts and data outages were noted concerns and were attributed to Wifi connectivity challenges and signal noise in the context of patient ambulation [11].

More recently, Downey et al [12] investigated the reliability of a wireless wearable patch, SensiumVitals, to monitor vital signs (ie, temperature, heart rate, respiration rate) continuously from patients (n=51) following major elective general surgery. Nurses’ manual vital sign observations were compared against paired instances of SensiumVitals biometric measurements. A median of 19 sets of manual measurements was captured for each patient, for a total of 1135 observation sets of paired comparisons for analysis [12]. In contrast to the results of this study, the error between manual and continuous vital sign measurements in the study by Downey et al [12] did not fall within prespecified limits of agreement, as defined through clinical expert consensus. Wide error distributions were again attributed to patient ambulation and related signal artifact, as well as possible human error during various manual vital sign measurements.

Data from these pilot studies support that wearable technologies capture continuous vital sign measurements from hospitalized ambulatory patients with varying degrees of accuracy. Whether prespecified levels of agreement with a reference standard are met, signal artifact and other sources of error, such as human error, pose challenges to validation of wearable sensor technologies in real-world clinical settings. Although our comparison of the Vitaliti CVSM to a continuous invasive reference (in an ICU) met strict ISO prespecified limits of agreement [16], our study patients were necessarily restricted to their hospital bed while undergoing cNIBP monitoring with an arterial catheter. Hence, in this environment, excessive patient motion and human measurement did not pose major challenges. The compliance of the Vitaliti CVSM with rigorous ISO standards [16] in a complex ICU environment is nonetheless promising; further validation testing in ambulatory patients is required.

A few studies have also examined wearable biosensor user acceptance from the patient perspective. In the pilot study of the ViSi Mobile and HealthPatch systems by Weenk et al [11], user experience was captured through semistructured interviews after patients had worn these devices for 2 to 3 consecutive days. Thematic content analysis revealed largely positive experiences, with most patients reporting that the monitoring devices were reassuring for them because nurses could monitor them from a distance [11]. Most also felt that these sensors did not encumber their personal care activities (eg, dressing, bathing). Good device adhesive and small sensor size were also noted as factors that were important to patients with respect to wearability of several device components [11]. Wearability of the Visi Mobile system was reported by several patients to be impacted negatively by the size and weight of the wristwatch component, numerous cables, as well as short battery life [11].

In a reactive post hoc analysis report, Harsha et al [24] examined challenges with implementing continuous oximetry monitoring in the VItal siGns monItoring with continuous puLse oximetry And wireless cliNiCian notification aftEr surgery (VIGILANCE) study (n= 2049), a randomized controlled trial testing the effectiveness of the Nellcor Oxinet III system (Covidien, Mansfield, MA) for continuous pulse oximetry (CPOX) monitoring on the incidence of postoperative respiratory complications among noncardiac surgery patients. VIGILANCE investigators found that 10.68% of patients withdrew from CPOX monitoring before intervention completion. Analysis of trial case report forms found a number of reasons for patient nonadherence, including obtrusiveness of the CPOX cables, an uncomfortable SpO_2_ probe, restrictions to ambulation, and device-related agitation of carpal tunnel syndrome [24].

In contrast to the usability assessments by Weenk et al [11] and Harsha et al [24], our examination of patient acceptance of the Vitaliti CVSM was in the context of a controlled measurement study, rather than in the context of live clinical system deployment where patients were ambulating on hospital wards and engaged in personal care activities. Although context differed, our results corroborate that patients value unobtrusive and comfortable sensor components. Our results also corroborate that patients are impacted negatively by technology features that are experienced as cumbersome or that require continual repositioning or reapplication.

### Strengths

Strengths of this study include the validation of the Vitaliti CVSM in the context of ISO standards for cNIBP measurement [16], as well as rigorous methods and planned approaches to post hoc signal analyses in order to ensure high data quality. Conduct of this study in a complex ICU setting also required the interconnection of numerous pieces of technology with operational independence to achieve time-matched cNIBP data sets for comparison of the Vitaliti CVSM with a gold-standard arterial catheter reference. An additional strength, therefore, was our plan to oversample to compensate for anticipated data losses due to technical problems and inevitable changes to patient hemodynamic status in an ICU setting.

### Limitations

Although the ISO standard [16] is rigorous from a measurement perspective, a limitation it imposed was the restriction of study participants to static (seated) and supine positions. Patients were also confined to bed while undergoing invasive cNIBP monitoring, thereby limiting the generalizability of our results to nonambulatory environments. It should also be noted that, in a complex ICU setting, this investigation could not span the full duration of the calibration period for the Vitaliti CVSM, which is 24 hours (Multimedia Appendix 1). Rather, this study was limited to evaluation of the device during a very short validation period after calibration (ie, that commenced within 2 minutes after calibration and that lasted for a short duration of time). For practical reasons within an acute ICU setting, we had to set up our equipment and take our BP measurements on each participant as expediently as possible to minimize disruption to nursing and medical care by virtue of the presence of our study team and related equipment. This timing precluded possible BP variations from calibration values that may otherwise be observed during a full 24-hour calibration period. Hence, results of this study cannot be applied to the BP accuracy of the Vitaliti CVSM over a 24-hour period with BP variations that may be normal, including individual readings that may vary considerably from calibration values. Future research should incorporate evaluation over the full calibration period of the device.

Our results for patients in the static and supine positions are within the clinically allowable tolerances for accuracy according to ISO. As such, claims regarding the accuracy of Vitaliti cNIBP can only be made at this point in the context of accuracy requirements set forth by Health Canada and the FDA. More research will be required to examine the accuracy of Vitaliti cNIBP measurements according to international standards, such as those set forth by the European Society of Hypertension [23] and the British Hypertension Society [25]. Examination of the accuracy of the Vitaliti cNIBP measurement according to these standards was not within the scope of this study.

Finally, patients enrolled in this study were postsurgical cardiac ICU patients given the requirement to compare the Vitalilti CVSM to an invasive gold-standard arterial catheter. The hemodynamic profile of patients in the postsurgical cardiac ICU typically features a greater degree of variability than seen in other populations in the early postoperative period. Moreover, some patients may experience atrial fibrillation following cardiac surgery. It should therefore be recognized that the included sample is not representative of all postsurgical patient populations—particularly those patients who undergo noncardiac and same day surgeries.

### Conclusions

Wearable RAM technologies that enable continuous acquisition of physiologic data from biosensors have the potential to transform postoperative care. This study found that one such wearable technology, Cloud DX’s Vitaliti CVSM, demonstrated cNIBP measurement in compliance with ISO 81060-2:2018 standards [16] in the context of evaluation that commenced within 2 minutes of device calibration; this device was also well-received by patients in a postsurgical ICU setting. Future studies will examine the accuracy of the Vitaliti CVSM in ambulatory contexts for both cardiac and noncardiac surgery patients, with attention to assessment of the impact of excessive patient motion on data artifacts and signal quality. The Vitaliti CVSM will also be evaluated longitudinally as part of a postoperative remote patient monitoring solution both in hospital and while patients are recovering at home for up to 30 days following surgery. This work will feature intensive focus on the use of derived vital metrics and high-fidelity physiological data collected with the Vitaliti CVSM in order to develop predictive models with machine learning. The aim of these predictive models will be to identify early signs of postoperative complications in order to facilitate timely clinical interventions.

## Appendix

Multimedia Appendix 1 Vitaliti continuous vital signs monitor (CVSM): device features and clinical workflow.

Multimedia Appendix 2 Vitaliti Patient Feedback Questionnaire.

## Abbreviations

AAMI: Association for the Advancement of Medical Instrumentation
BP: blood pressure
cNIBP: continuous non-invasive blood pressure
CPOX: continuous pulse oximetry
CVSM: continuous vital signs monitor
DBP: diastolic blood pressure
ECG: electrocardiogram
FDA: Food and Drug Administration
ICU: intensive care unit
ISO: International Organization for Standardization
LED: light emitting diode
PHRI: Population Health Research Institute
RAM: remote automated monitoring
SBP: systolic blood pressure
SpO_2_: blood oxygen saturation
VIGILANCE: VItal siGns monItoring with continuous puLse oximetry And wireless cliNiCian notification aftEr surgery

## References

[1] Aseni P, Orsenigo S, Storti E, Pulici M, Arlati S (2019). Current concepts of perioperative monitoring in high-risk surgical patients: a review. Patient Saf Surg.

[2] McGillion MH, Duceppe E, Allan K, Marcucci M, Yang S, Johnson AP, Ross-Howe S, Peter E, Scott T, Ouellette C, Henry S, Le Manach Y, Paré G, Downey B, Carroll SL, Mills J, Turner A, Clyne W, Dvirnik N, Mierdel S, Poole L, Nelson M, Harvey V, Good A, Pettit S, Sanchez K, Harsha P, Mohajer D, Ponnambalam S, Bhavnani S, Lamy A, Whitlock R, Devereaux P, PROTECT Network Investigators (2018). Postoperative remote automated monitoring: need for and state of the science. Can J Cardiol.

[3] Efthymiou CA, O'Regan DJ (2011). Postdischarge complications: what exactly happens when the patient goes home?. Interact Cardiovasc Thorac Surg.

[4] Leuvan CHV, Mitchell I (2008). Missed opportunities? An observational study of vital sign measurements. Crit Care Resusc.

[5] McGain F, Cretikos MA, Jones D, Van Dyk S, Buist MD, Opdam H, Pellegrino V, Robertson MS, Bellomo R (2008). Documentation of clinical review and vital signs after major surgery. Med J Aust.

[6] Sun Z, Sessler DI, Dalton JE, Devereaux PJ, Shahinyan A, Naylor AJ, Hutcherson MT, Finnegan PS, Tandon V, Darvish-Kazem S, Chugh S, Alzayer H, Kurz A (2015). Postoperative hypoxemia is common and persistent: a prospective blinded observational study. Anesth Analg.

[7] Sessler DI, Saugel B (2019). Beyond 'failure to rescue': the time has come for continuous ward monitoring. Br J Anaesth.

[8] Turan A, Chang C, Cohen B, Saasouh W, Essber H, Yang D, Ma C, Hovsepyan K, Khanna AK, Vitale J, Shah A, Ruetzler K, Maheshwari K, Sessler DI (2019). Incidence, severity, and detection of blood pressure perturbations after abdominal surgery: a prospective blinded observational study. Anesthesiology.

[9] Joshi M, Ashrafian H, Aufegger L, Khan S, Arora S, Cooke G, Darzi A (2019). Wearable sensors to improve detection of patient deterioration. Expert Rev Med Devices.

[10] Khanna AK, Hoppe P, Saugel B (2019). Automated continuous noninvasive ward monitoring: future directions and challenges. Crit Care.

[11] Weenk M, van Goor H, Frietman B, Engelen LJ, van Laarhoven CJ, Smit J, Bredie SJ, van de Belt TH (2017). Continuous monitoring of vital signs using wearable devices on the general ward: pilot study. JMIR Mhealth Uhealth.

[12] Downey C, Ng S, Jayne D, Wong D (2019). Reliability of a wearable wireless patch for continuous remote monitoring of vital signs in patients recovering from major surgery: a clinical validation study from the TRaCINg trial. BMJ Open.

[13] Ogedegbe G, Pickering T (2010). Principles and techniques of blood pressure measurement. Cardiology Clinics.

[14] Sahu D, Bhaskaran M (2010). Palpatory method of measuring diastolic blood pressure. J Anaesthesiol Clin Pharmacol.

[15] Meidert AS, Saugel B (2018). Techniques for non-invasive monitoring of arterial blood pressure. Front. Med.

[16] ISO 81060-2:2018 - Non-invasive sphygmomanometers - Part 2: Clinical investigation of intermittent automated measurement type. International Organization for Standardization.

[17] Vitaliti. CloudDX.

[18] Bland JM, Altman DG (2016). Measuring agreement in method comparison studies. Stat Methods Med Res.

[19] MediCollector.

[20] (2016). Applying Human Factors and Usability Engineering to Medical Devices: Guidance for Industry and Food and Drug Administration Staff. US Food and Drug Administration.

[21] Vankipuram M, McMahon S, Fleury J (2012). ReadySteady: app for accelerometer-based activity monitoring and wellness-motivation feedback system for older adults. AMIA Annu Symp Proc.

[22] Chen W, Kobayashi T, Ichikawa S, Takeuchi Y, Togawa T (2000). Continuous estimation of systolic blood pressure using the pulse arrival time and intermittent calibration. Med. Biol. Eng. Comput.

[23] O'Brien E, Atkins N, Stergiou G, Karpettas N, Parati G, Asmar R, Imai Y, Wang J, Mengden T, Shennan A, Working Group on Blood Pressure Monitoring of the European Society of Hypertension (2010). European Society of Hypertension International Protocol revision 2010 for the validation of blood pressure measuring devices in adults. Blood Press Monit.

[24] Harsha P, Paul JE, Chong MA, Buckley N, Tidy A, Clarke A, Buckley D, Sirko Z, Vanniyasingam T, Walsh J, McGillion M, Thabane L (2019). Challenges With Continuous Pulse Oximetry Monitoring and Wireless Clinician Notification Systems After Surgery: Reactive Analysis of a Randomized Controlled Trial. JMIR Med Inform.

[25] O'Brien E, Petrie J, Littler W, de Swiet M, Padfield PL, O'Malley K, Jamieson M, Altman D, Bland M, Atkins N (1990). The British Hypertension Society protocol for the evaluation of automated and semi-automated blood pressure measuring devices with special reference to ambulatory systems. J Hypertens.

