# Supplementary material for: Continuous Noninvasive Remote Automated Blood Pressure Monitoring With Novel Wearable Technology: A Preliminary Validation Study
Source: JMIR Mhealth Uhealth. 2022 Feb 28;10(2):e24916. doi: 10.2196/24916 (PMC8922156; doi:10.2196/24916)
Supplement: Multimedia Appendix 1 [file mhealth_v10i2e24916_app1.docx]

**Multimedia Appendix A:** **Vitaliti™ Continuous Vital Signs Monitor (CVSM) – Device Features and Clinical Workflow**

**Features**

- Device is paired with companion software for cloud-based computing; it also features a tablet-based, user-friendly interface (Figure 1), Bluetooth Low Energy and WiFi for communication with smartphones, tablets, and personal computers.
- Device operates for up to 52 hours on a single charge during active transmission and can be fully charged in 1 hour.
- Maintains an Ingress Protection rating of 22 to support infection control routines for cleaning and disinfecting and is intended for use in-hospital and home healthcare environments.
- The detachable earpiece, for the measurement of core temperature and continuous non-invasive blood pressure, has a semi-flexible hinge that holds the light emitting diode sensor in place to ensure high signal quality.
- The Vitaliti™ CVSM has been designed with an extensible hardware architecture; this will allow alterative senor accessories to be mounted on the forehead or finger for those patients who could not accommodate placement of a sensor in the ear.
- Device contains numerous accelerometer and gyroscope sensors to capture body position and proximity sensing, to detect usage and optimize power saving. Vitaliti™ contains onboard storage for up to 6 hours of continuous capture of derived vital metrics, as well as 15 high-fidelity physiological signals.

**Workflow**

- Device is first calibrated with a reference blood pressure and then utilizes an analysis of the electrocardiogram and photoplethysmography to determine continuously changing fluctuations.
- Device calibrations are performed every 24 hours against a reference blood pressure standard. Important to the algorithm derivation is pulse transit time, which measures the propagation time between the electrocardiogram beat and the pulse waveform.
