# Supplementary material for: Continuous Noninvasive Remote Automated Blood Pressure Monitoring With Novel Wearable Technology: A Preliminary Validation Study
Source: JMIR Mhealth Uhealth. 2022 Feb 28;10(2):e24916. doi: 10.2196/24916 (PMC8922156; doi:10.2196/24916)
Supplement: Multimedia Appendix 2 [file mhealth_v10i2e24916_app2.docx]

**Multimedia Appendix B: Vitaliti Patient Feedback Questionnaire**

| Question | Strongly Disagree | Disagree | Neither Agree or Disagree | Agree | Strongly Agree |
| --- | --- | --- | --- | --- | --- |
| 1) The device was easily placed around my neck. |  |  |  |  |  |
| 2) The device is comfortable while sitting. |  |  |  |  |  |
| 3) The device is comfortable while standing. |  |  |  |  |  |
| 4) The device was comfortable while lying down. |  |  |  |  |  |
| 5) The weight of the device was reasonable to wear for the duration of the testing period. |  |  |  |  |  |
| 6) I like the colour of the device. |  |  |  |  |  |
| 7) The collar and ear piece was visually appealing. |  |  |  |  |  |
| 8) The ECG leads were easily applied. |  |  |  |  |  |
| 9) The ear piece was easy to put on. |  |  |  |  |  |
| 10) The ear piece was easy to take off. |  |  |  |  |  |
| 11) The ear piece was comfortable to wear. |  |  |  |  |  |
| 12) The ear piece remained in place throughout the test. |  |  |  |  |  |
| 13) I would recommend the device to my friends and family. |  |  |  |  |  |
